# Paget's Disease of the Breast in a Patient with Amyopathic Dermatomyositis

**DOI:** 10.1155/2012/515691

**Published:** 2012-09-19

**Authors:** Sirin Yasar, Gunay Gurleyik, Yesim Sabuncuoglu, Ali Aktekin, Bulent Yasar, Zehra A. Serdar

**Affiliations:** ^1^Department of Dermatology, Haydarpaşa Numune Teaching and Research Hospital, 34668 Istanbul, Turkey; ^2^Department of Surgery, Haydarpaşa Numune Teaching and Research Hospital, 34668 Istanbul, Turkey; ^3^Department of Internal Medicine, Haydarpaşa Numune Teaching and Research Hospital, 34668 Istanbul, Turkey

## Abstract

Amyopathic dermatomyositis (AD) can be a part of paraneoplastic syndrome of an underlying malignancy. Paget's disease is a rare form of breast cancer. We present a very rare case of Paget's disease associated with AD. Paget's disease has been diagnosed in a patient with AD who is under surveillance of dermatology department. The patient has undergone central lumpectomy with removal of the nipple-areola complex and sentinel lymph node biopsy. Surgical margins after lumpectomy and sentinel node biopsy were negative. The whole breast irradiation was performed after surgery. The patient receives medical treatment for AD of which lesions regressed in 1 year during the follow-up period. This is a very rare case of Paget's disease diagnosed in a patient with AD. Female patients with dermatomyositis have been absolutely recommended to undergo screening for breast and gynaecological malignancies. AD may be an early finding of primary or recurrent malignancy of the breast.

## 1. Introduction 

Amyopathic dermatomyositis (AD) or “dermatomyositis sine myositis” is an autoimmune disease accompanied with heliotrope rash, Gottron's papules, and cuticular changes in nails and histopathologic skin findings of dermatomyositis. Similar to classical dermatomyositis, AD can be a part of paraneoplastic syndrome of an underlying malignancy [1]. Goyal and Nousari [2] have reported cases of AD accompanied with breast carcinoma. Meanwhile Paget's disease of the breast in a patient with AD has not been reported.

 Paget's disease is a rare presentation of breast cancer, most of patients present with eczema or ulceration of the nipple. The diagnosis can be obtained by punch or wedge biopsy. The Paget's cells can be determined by immunohistochemical studies [3]. 

In this paper, we present Paget's disease of the breast which has been diagnosed in a patient with AD. 

## 2. Case

**Figure 1:**
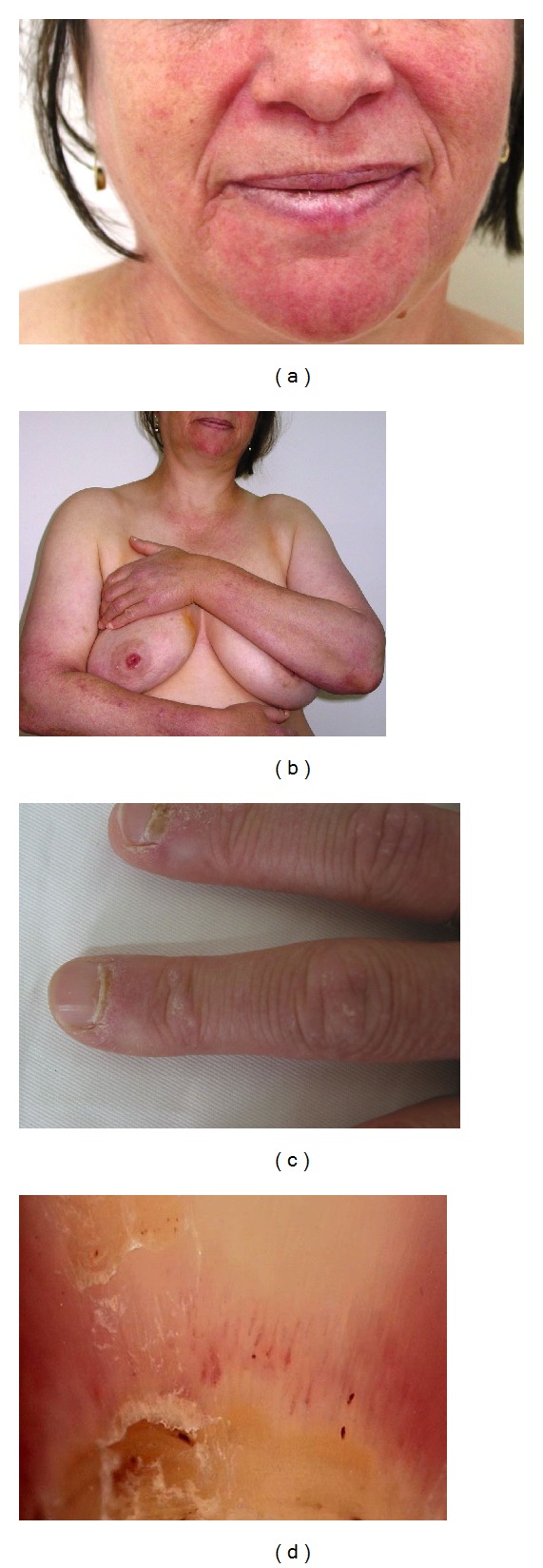
(a) Erythematous-violaceous plaques on face, (b) arms, and sun-exposed regions, and (c) periungual telangiectasias were shown via (d) capillaroscopy.

The 57-year-old female patient applied with complaints of skin rashes and itching on her face, arms, and chest aggravated during the summer months. These skin lesions and itching were unresponsive to treatment of antihistamines and topical steroids. She also had livedoid, erythematous, oedematous patches around her eyes, and erythematous patchy lesions on the sunlight-exposed body parts of face, interscapular region of back, V-region of anterior face of chest, and extensor face of upper extremities. There were also erythematous papules localized on the metacarpophalangeal and interphalangeal joints of bilateral hand dorsum beside periungual telangiectasias in bilateral hand fingers. The capillaroscopy of periungual telangiectasias encountered dilatation in capillary loops (Figures 1(a), 1(b), 1(c), and 1(d)). 

**Figure 2:**
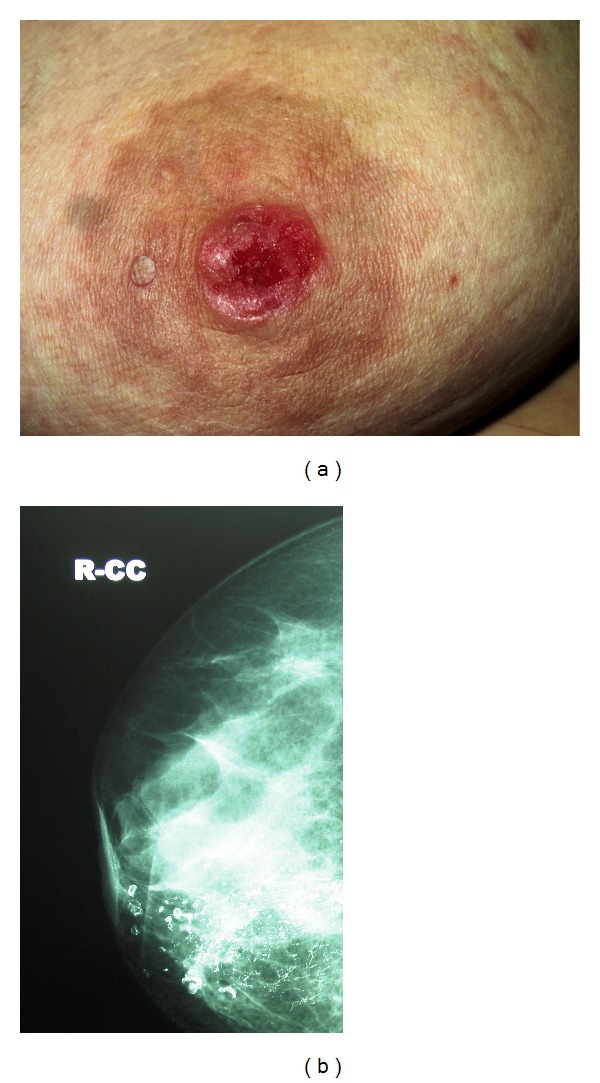
(a) An ulcerated lesion on the right nipple. (b) Pleomorphic and linear microcalcifications on the mammogram.

The physical examination has shown no proximal muscle weakness. Laboratory serologic examination was positive for antinuclear antibody (ANA) in 1/100 dilution in homogeneous pattern; but extractable nuclear antigens (ENA) profile including Anti-Jo-1 and Anti-Mi-2 was negative. Her muscle activity during electromyography (EMG) monitoring was also within normal limits. The skin biopsy of the right arm revealed basket-weave hyperkeratosis through the epidermis, atrophy, vacuolar degeneration of the basal lamina, papillary dermal oedema, and perivascular lymphocytic infiltration demonstrating superficial perivascular dermatitis. It was evaluated in favour of dermatomyositis, while excluding subacute lupus erythematosis. In the direct immunofluorescence examination, irregular staining groups for IgM and IgA were monitored at the tips of papillae in the basal lamina (Figures 2(a) and 2(b)). The lupus-band test obtained from sun-protected nonlesion skin was negative. The patient was diagnosed as “amyopathic dermatomyositis.” 

**Figure 3:**
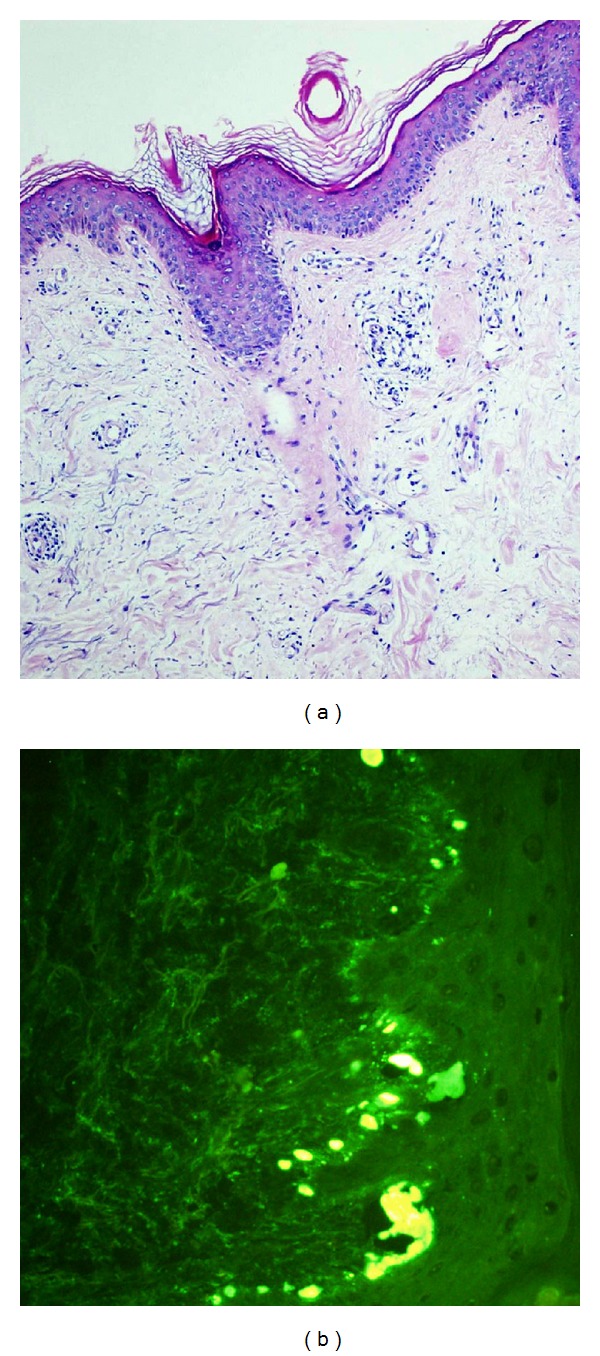
(a) Superficial perivascular dermatitis; basket-weave hyperkeratosis through the epidermis, slight atrophy, vacuolar degeneration of the basal lamina, papillary dermal oedema, and perivascular lymphocytic infiltration (H&E ×200). (b) In the direct immunofluorescence examination, irregular staining groups for IgM and IgA were monitored at the tips of papillae of the basal lamina.

**Figure 4:**
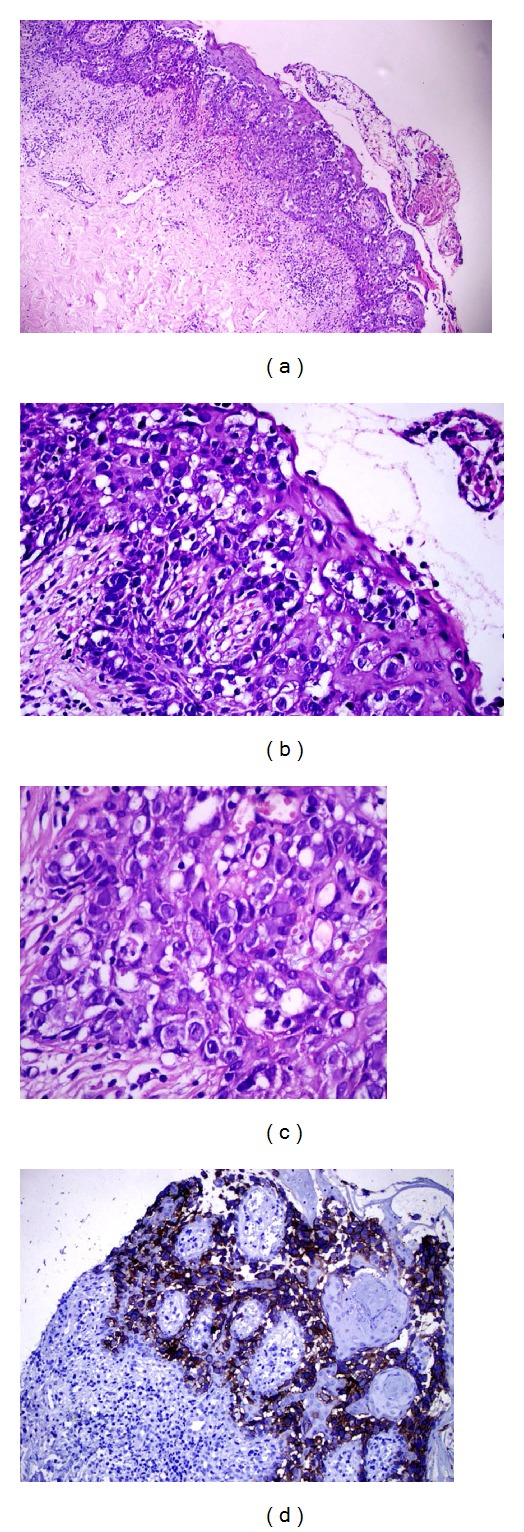
Atypical cellular proliferation with clear cytoplasm and large hyperchromatic nuclei arrangement in the basal lamina of the epidermis and epidermotropism towards the upper rows of the epidermis (H&E (a) ×100, (b) ×200, and (c) ×400). (d) Positive staining for epithelial membrane antigen (EMA) in the immunohistochemical staining (×400).

On the other hand, she had erosive lesions on her right nipple for the recent 6 months. There were ulceration and eczematous changes on the right nipple-areola complex (NAC) (Figure 3(a)). Subareolar pleomorphic calcifications were detected with mammography (Figure 3(b)). However ultrasound did not demonstrate any lesion. In the magnetic resonance imaging of right breast, the focal progressive contrast involvement in the right nipple was compatible with Paget's disease. Punch biopsy from her right nipple detected atypical cellular proliferation with clear cytoplasm and large hyperchromatic nuclei arranged in the basal lamina of the epidermis and also epidermotropism towards the upper rows of the epidermis. Immunohistochemical staining showed positive staining for carcinoembryonic antigen (CEA), C erb B2, and epithelial membrane antigen (EMA) (Figures 4(a), 4(b), 4(c), and 4(d)). The lesion in the right nipple was diagnosed as Paget's disease according to the clinical, radiological, and histopathological findings. Atypical ductal hyperplasia and microcalcifications were detected by histopathological examination after core biopsy. The patient has undergone central lumpectomy with removal of the NAC, and sentinel lymph node biopsy. Surgical margins after lumpectomy and sentinel biopsy were negative. Pathological examinations of excised breast tissue demonstrated high grade, comedo type (DIN III) of in situ ductal carcinoma (DCIS), lobular cancerisation, and ductal pagetoid invasion. Oestrogen and progesterone receptors were negative. The whole breast irradiation was performed after surgery. The patient was also initiated medical treatment with hydroxychloroquine sulphate 200 mg 2 × 1 and topical mid potency steroid cream with sun-protection factor. The dermatomyositis lesions regressed in 1 year during the follow-up period.

## 3. Discussion

Amyopathic dermatomyositis has been first described by Pearson in 1963. It is a phenomenon accompanied with characteristic clinical findings without inflammatory myopathy [1, 4]. Similarly, AD may be accompanied with malignancies like classical dermatomyositis. The malignancy rate accompanied with dermatomyositis has been reported as 15–30% in adults. The malignant change in our patient as Paget's disease is a rare pathology of the nipple-areola complex (NAC) associated with an underlying in situ or invasive carcinoma. Eczematous changes accompanied with pain and itching in the NAC are monitored in patients with Paget's disease [3, 5]. Paraneoplastic dermatomyositis may develop following the malignancies, as well as it may occur previous to or after malignancies. Lesions may regress after treatment of the malignant tumour [2, 4]. In our case, Paget's disease with DCIS had been diagnosed after one year of the clinical findings of AD. Kuo et al. [6] have reported 803 cases that 13.8% of these patients had breast cancer. The cancer diagnosis was generally made (64%) after dermatomyositis. Hashimoto et al. [5] have reported a case with dermatomyositis recruited following resection of breast cancer and exacerbation in clinical findings of dermatomyositis due to ovarian cancer. Patients with dermatomyositis associated with breast cancer should be controlled with respect to ovarian carcinoma when clinical findings of dermatomyositis were relapsed. In our case, no pathological findings were found by transvaginal ultrasound in ovarian cancer screening. Osako et al. [7] have reported a case of relapsing of dermatomyositis that amyopathic dermatomyositis regressed after the resection of breast cancer. It has been emphasized that dermatomyositis lesions may be an important indicator in recurrence of breast cancer. Mebazaa et al. [8] have evaluated patients with dermatomyositis associated with breast cancer. The clinical picture of dermatomyositis has regressed due to the treatment of breast cancer in 9 of 13 patients in these series. Song et al. [9] have reported improvement of the dermatomyositis symptoms during 9–15 months of followup after the surgery.

Seventy percent of patients with dermatomyositis are evaluated as paraneoplastic syndrome. In our patient with AD, Paget's disease and high grade comedo type of DCIS were diagnosed. After excision of NAC and central wide local excision of breast tissue with microscopically clear margins, and whole breast irradiation, the remarkable regression of dermatomyositis lesions during the 1-year followup allows us to consider that it occurs as a paraneoplastic phenomenon. 

In conclusion, dermatomyositis may accompany a paraneoplastic syndrome or may occur during the course of a malignancy. The accompaniment of Paget's disease of the nipple with AD is a very rare event. Female patients with dermatomyositis above 40-year-old have been absolutely recommended to undergo breast and gynaecological examination by clinical, laboratory, and imaging modalities. AD may be an early finding of primary or recurrent malignancy of the breast and the ovary, including Paget's disease of the breast.
